# Fundamentals of genomic epidemiology, lessons learned from the coronavirus disease 2019 (COVID-19) pandemic, and new directions

**DOI:** 10.1017/ash.2021.222

**Published:** 2021-12-07

**Authors:** Denis Jacob Machado, Richard Allen White, Janice Kofsky, Daniel A. Janies

**Affiliations:** 1 University of North Carolina at Charlotte, College of Computing and Informatics, Department of Bioinformatics and Genomics, Charlotte, North Carolina; 2 University of North Carolina at Charlotte, North Carolina Research Campus (NCRC), Kannapolis, North Carolina

## Abstract

The coronavirus disease 2019 (COVID-19) pandemic was one of the significant causes of death worldwide in 2020. The disease is caused by severe acute coronavirus syndrome (SARS) coronavirus 2 (SARS-CoV-2), an RNA virus of the subfamily *Orthocoronavirinae* related to 2 other clinically relevant coronaviruses, SARS-CoV and MERS-CoV. Like other coronaviruses and several other viruses, SARS-CoV-2 originated in bats. However, unlike other coronaviruses, SARS-CoV-2 resulted in a devastating pandemic. The SARS-CoV-2 pandemic rages on due to viral evolution that leads to more transmissible and immune evasive variants. Technology such as genomic sequencing has driven the shift from syndromic to molecular epidemiology and promises better understanding of variants. The COVID-19 pandemic has exposed critical impediments that must be addressed to develop the science of pandemics. Much of the progress is being applied in the developed world. However, barriers to the use of molecular epidemiology in low- and middle-income countries (LMICs) remain, including lack of logistics for equipment and reagents and lack of training in analysis. We review the molecular epidemiology literature to understand its origins from the SARS epidemic (2002–2003) through influenza events and the current COVID-19 pandemic. We advocate for improved genomic surveillance of SARS-CoV and understanding the pathogen diversity in potential zoonotic hosts. This work will require training in phylogenetic and high-performance computing to improve analyses of the origin and spread of pathogens. The overarching goals are to understand and abate zoonosis risk through interdisciplinary collaboration and lowering logistical barriers.

## How did genomic epidemiology become what it is?

**Fig. 1.**
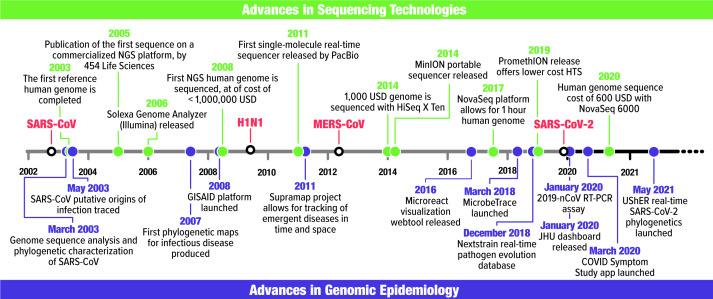
Timeline of major events in sequencing technology (green) and genomic epidemiology (purple) alongside the first recorded occurrence of SARS-CoV, H1N1-2009, MERS-CoV, and SARS-CoV-2 in humans. Associated references can be found in Supplementary Table 1.

Genomic epidemiology stems from molecular epidemiology, which uses evidence ranging from gel electrophoresis to multilocus sequence typing to study the origins and spread of pathogenic microorganisms. Janies et al^1^ reviewed the history of molecular epidemiology and compared it with syndromic epidemiology. Here, we focus on recent advances toward genomic epidemiology (Fig. 1), which includes genomic sequencing combined with rapid data sharing as enabled by the Internet. In 2002–2003, the severe acute respiratory syndrome coronavirus (SARS-CoV) was the first infectious disease for which scientists shared software and pathogen genetic data over the Internet to rapidly respond to the disease. Thereafter, genomic epidemiology was solidified by responses to H5N1, H1N1-2009, and other strains of influenza such as H7N9^2^ and expanded to respond to foodborne and sexually transmitted diseases.^3–5^

The first SARS-CoV genome was shared after publication^6,7^ on National Center for Biotechnology Information’s (NCBI) GenBank website, which was customary. Meanwhile, dashboards, graphs, and maps emerged to track cases over time and space.^8^ Janies et al^9,10^ combined genomic and geographic data for SARS-CoV and H5N1 influenza, respectively, being the first to project phylogenies onto a virtual globe. Janies et al^11^ used Keyhole Markup Language (KML) to develop Supramap, which facilitates geographic mapping of phylogenies. Supramap allowed hypothesis testing ranging from the host and geographic origins of pathogens^12^ to tracing mutations that conferred drug resistance or host switching.^13,14^ Limitations of computing large data sets, coupled with a preference for sharing data after publication, resulted in a greater turnaround between data acquisition and results than occurs today. However, these conditions did not impede a hypothesis-driven field with value to decision makers, as demonstrated in a 2007 congressional hearing.^15^

In the 2000s, some genomes were sequenced for respiratory pathogens such as H1N1-2009. However, even SARS-CoV genomes were not always sequenced completely, and sequences were released gradually.^9^ This changed due to factors such as new DNA sequencing technologies.

## How did advances in sequencing technology reshape genomic epidemiology?

Current genomic epidemiology of infectious diseases originated in response to the SARS-CoV epidemic.^16^ Sequencing the SARS-CoV genome was instrumental in recognizing it as a novel coronavirus associated with HCoV-OC43 and HCoV-229E.^6,7^ Researchers combined genomic and epidemiological data to trace the genotypic variation of the viral transmission paths between 2002 and 2003.^17,18^ However, today’s genomic surveillance evolved with the advance of high-throughput sequencing (HTS) (Fig. 1).

**Fig. 2.**
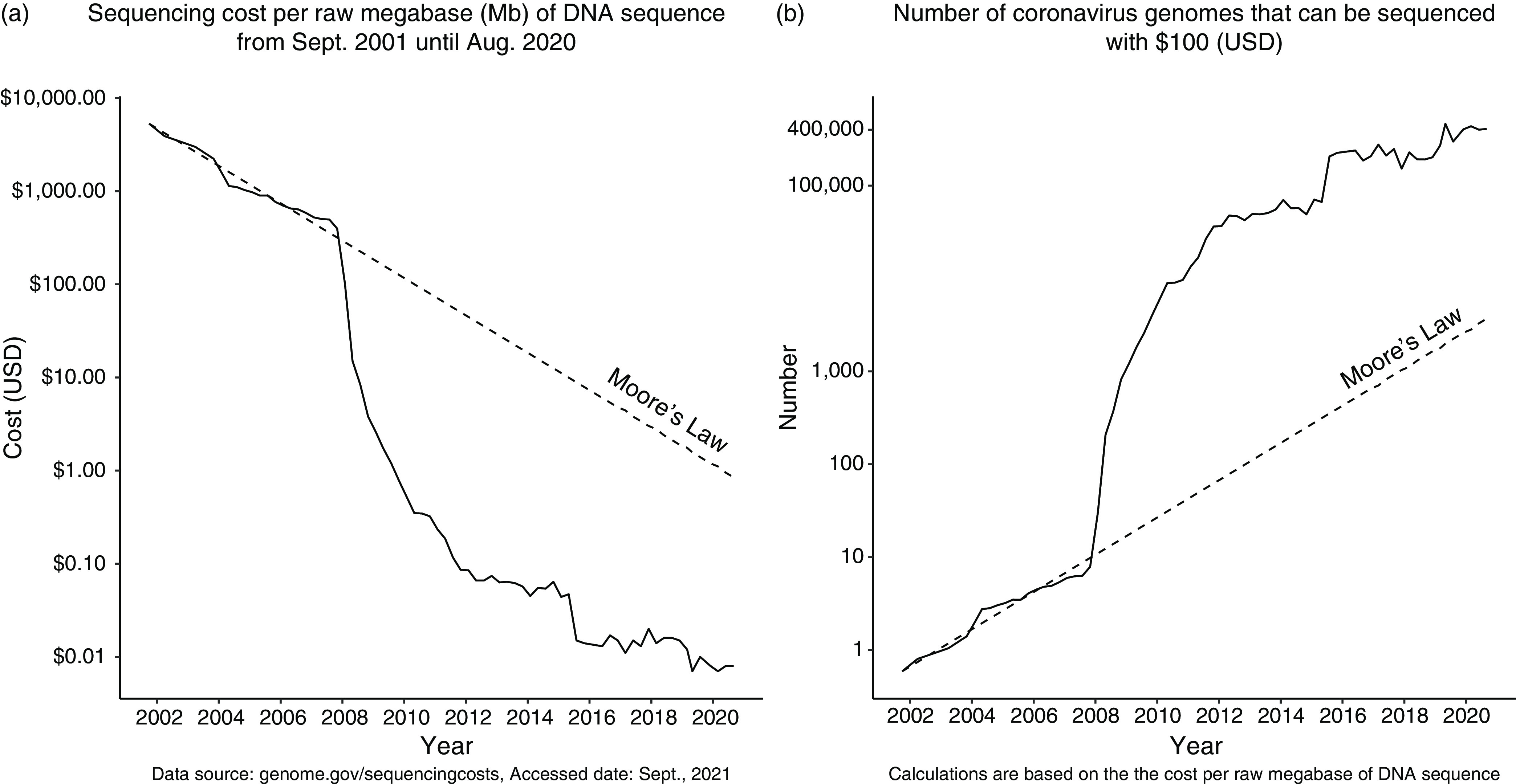
The increasing feasibility of sequencing complete coronavirus genomes. (a) Sequencing cost per raw megabase of DNA sequence from September 2001 until August 2020 (data source: genome.gov/sequencingcosts, access date: September 2021). (b) Number of complete coronavirus genomes that can be sequenced with USD 100, assuming a genome size of 32 Kbp. These cost estimates do not consider sampling, storage, consumables, equipment, and staff costs. These plots use a logarithmic scale.

Reuter et al^19^ summarized HTS history until 2015 and Pérez-Losada^20^ reviewed recent HTS advances. We focus on the sequence cost variation per raw megabase between 2001 and 2020^21^ (Fig. 2a) to illustrate the increasing feasibility of sequencing coronavirus genomes (Fig. 2b**)**. Considering raw nucleotide sequencing cost, US$100 was not sufficient to sequence one coronavirus genome in 2020, but $100 it would cover >400,000 genomes in 2020.

## What are coronaviruses?

Coronaviruses correspond to the four genera of the subfamily *Orthocoronavirinae*. *Gammacoronavirus* (GammaCoVs) and *Deltacoronavirus* (DeltaCoVs) mainly infect birds and rarely infect mammals.^22,23^ *Alphacoronavirus* (AlphaCoVs) and *Betacoronavirus* (BetaCoVs) originated from Chiroptera (bats) and are often found in other mammals, including humans.^24^

**Fig. 3.**
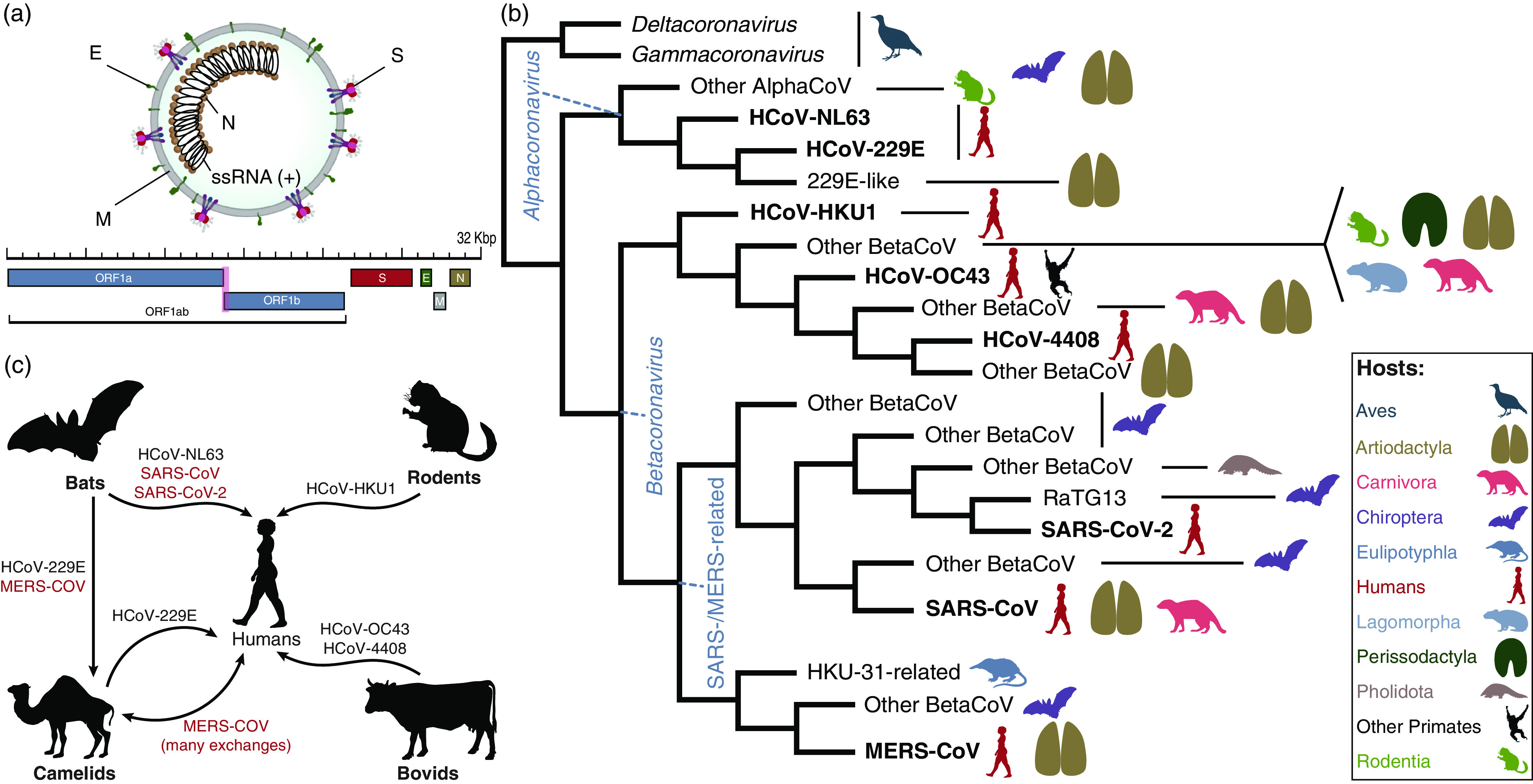
Fundamental evolution of coronaviruses based on Machado et al.^49^ (a) Virion and genome structure. The genomic regions indicated in the figure do not represent all the genes in the coronavirus genome, but the genes that are shared among the different genera of *Orthocoronavirinae* and that were analyzed by Machado et al.^49^ Note. E, envelope small membrane protein; M, membrane protein; N, nucleoprotein; S, spike glycoprotein. (b) Summarized cladogram from Machado et al.^49^ The original cladogram contained 2,006 terminals corresponding to unique coronavirus genomes. Terminals indicating the eight species of human coronaviruses (HCoVs) are in bold. (c) Hosts involved in the emergence of all human coronaviruses, including SARS-CoV-2. The HCoVs of special concern to human health (SARS-CoV, MERS-CoV, and SARS-CoV-2) are shown in red. The flow chart indicates that HCoV-NL63, SARS-CoV, and SARS-CoV-2 originated from bat-hosted coronaviruses. Bats were also key to the emergence of MERS-CoV in camels and humans. HCoV-229E, HCoV-HKU1, and HCoV-OC43 originated from viruses hosted in artiodactyls, rodents, and bovids, respectively. All silhouettes were downloaded from PhyloPic (http://phylopic.org). The coronavirus vision structure was modified from https://commons.wikimedia.org/wiki/File:Coronavirus_virion_structure.svg. See Supplementary File 1 for detailed copyright and license information.

The coronavirus virion encapsulates one of the longest RNA virus genomes (27–32 kb),^25^ which has complex gene expression^26^ and variable gene content among genera (Fig. 3a).^27^

Coronavirus infections in domestic animals are economically significant.^28–30^ However, the episodic emergence of human coronaviruses (HCoVs) is a pressing concern because they cause infections in all age groups, often leading to respiratory or enteric diseases.^31^ Neurological illness or hepatitis is less frequent.^32^ The US Centers for Disease Control (CDC) website^33^ lists 7 HCoVs: 2 AlphaCoVs (HCoV-229E and HCoV-NL63) and 5 BetaCoVs (HCoV-OC43, HCoV-HKU1, SARS-CoV, MERS-CoV, and SARS-CoV-2). We added the human enteric coronavirus 4408 (HECV-4408) to the list because it was isolated from a child with acute gastroenteritis.^34^

## How did SARS-CoV-2 accelerate the growth of genomic epidemiology?

Coronaviruses were not deemed highly pathogenic to humans until the 2002 SARS-CoV outbreak.^35,36^ The dangers of HCoVs were made more evident by the 2012 outbreak of Middle East respiratory syndrome (MERS) coronavirus (MERS-CoV).^37^ Nevertheless, coronaviruses did not receive the current level of attention until the pandemic coronavirus disease 2019 (COVID-19), caused by SARS-CoV-2, was first reported in humans in Wuhan, China, in December 2019.^38^ However, Pekar et al^39^ inferred that the virus was present in Hubei approximately a month before. On March 11, 2020, the World Health Organization (WHO) declared a pandemic due to the spread of SARS-CoV-2.^38^ By October 14, 2021, COVID-19 had caused 4,863,818 deaths worldwide.^40^

Understanding the emergence and evolution of SARS-CoV-2 is vital to preventing future pandemics.^41^ The question can be divided into 3 components. First, was the virus purposefully manipulated? Several peer-reviewed publications have concluded that SARS-CoV-2 emerged naturally via zoonosis (see eg, Anderson et al,^42^ Liu et al^43^, and Holmes et al^44^). Moreover, previous serology data indicate natural human infections by bat-hosted, SARS-like viruses.^45^

Second, was SARS-CoV-2 an accidental release? If a naturally occurring virus was transported to a laboratory and humans were infected shortly thereafter, the virus may not have accumulated sufficient mutations to record its passage through controlled environments.^46^ However, no evidence indicates that SARS-CoV-2 was known to scientists before December 2019.^47,48^

Third, what is the natural source of SARS-CoV-2? The most comprehensive phylogenomic analysis of coronavirus^49^ (Fig. 3b) addressed the fundamental evolution of HCoVs (Fig. 3c) and showed that SARS-CoV-2 results from bat-hosted viruses infecting humans.^50^ SARS-CoV-2 finds its closest related bat-hosted coronaviruses in the subgenus *Sabercovirus*, a subgroup of SARS-related coronaviruses (SARSr-CoV) first identified in horseshoe bats (*Rhinophulus* spp).^51^ Bat-hosted viruses similar to SARS-CoV-2 were collected in the Yunnan province, >1,500 km away from Wuhan, but the hosts have a wide geographic range.^45,52,53^

Despite a confusing array of reports confirming^54–56^ and denying^57^ the origin of SARS-CoV-2 from pangolin (*Manis javanica*) hosts, pangolins are not involved in the lineage of SARS-CoV-2 that infected humans.^49^ This finding is similar to the emergence of SARS-CoV,^9^ which also infected humans from bat-hosted viruses without any need for intermediate hosts, including Himalayan palm civets (*Parguma larvata*) and raccoon dogs (*Nyctereutes procyonoides*).

## Are we sequencing SARS-CoV-2 genomes fast enough?

SARS-CoV-2 was identified on January 7, 2020. Three days later, its genome and metadata were shared via the Global Initiative on Sharing Avian Influenza Data (GISAID)^58^ EpiCoV database,^59^ before the first peer-reviewed article was published in February 2020.^60^

To put the SARS-CoV-2 genome sequencing speed into context, consider that SARS-CoV was first reported in November 2002, but its genome was publicly released in April 2003.^6^ The speed at which such data are released was changed by several forces, illustrated by Janies et al.^16^ In brief, the reasons include the increased feasibility of genome sequencing, the willingness to share data before publication, and the rise of the popular GISAID database, which credits submitting laboratories.

**Fig. 4.**
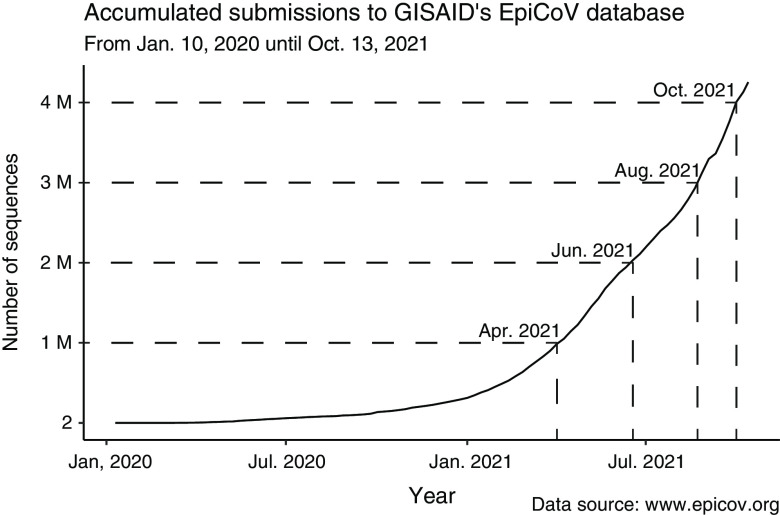
Progressive accumulation of 4,224,785 complete SARS-COV-2 genome sequences (>26 Kbp) submitted to the GISAID EpiCoV database (https://www.epicov.org/) between January 10, 2020, and October 13, 2021. These cost estimates do not consider sampling, storage, consumables, equipment, and staff costs (see eg, Schwarze et al^168^). Nevertheless, the price of raw nucleotide sequencing is a significant component of the cost of genome projects.

Figure 4 shows the accumulation of 4,224,785 complete SARS-CoV-2 genomes in EpiCoV between January 10, 2020, and October 13, 2021. The curve is far from reaching a plateau, indicating that we are not producing coronavirus genomes at total capacity. Efforts to sequence SARS-CoV-2 following international guidelines^61,62^ are welcome because these data inform epidemiological forecasts (eg, increased transmission efficiency of SARS-CoV-2 variants has led to projections of the rise of higher numbers of cases^63^).

Genomic sequencing generates a snapshot of a viral lineage in a place and time. When sequences are collected longitudinally, applications in genomic epidemiology and pandemic responses emerge, which we illustrate with 4 examples. First, profiling mutation fingerprints from the viral pangenome to individual infection quasi-species enables molecular contact tracing.^64^ Second, genomic sequencing informs the peptide mass fingerprinting (PMF) used to predict novel structures and find inhibitors for viral peptides,^65^ although results must be tested in randomized controlled trials^66^ to identify effective antivirals.^67,68^ Third, the data are used to model epidemic or pandemic size and severity.^63^ Fourth, viral sequences are fundamental for developing mRNA vaccines.^69^ For a review on current pitfalls and opportunities in applying HTS to SARS-CoV-2 genomes, see Chiara et al.^70^

As SARS-CoV-2 becomes endemic,^71,72^ sequencing demand will remain high. SARS-CoV-2 infections are decreasing as more people develop immunity through natural infection or vaccination.^73^ However, variants may evade infection and vaccine-induced antibodies,^74^ especially with infections occurring months after vaccination (ie, breakthrough infections).^75,76^ Given breakthrough infections, increased transmission of some variants, and the lack of full vaccination among eligible people, we can predict that SARS-CoV-2 will continue to evolve. Whether SARS-CoV-2 is evolving toward more severe or more benign COVID-19 phenotypes is a pressing research question for genomic epidemiology.

Effective countermeasures depend on understanding SARS-CoV-2 lineages, such as sampling variants for which phenotype is not fully understood^77^ and addressing sampling bias.^78^ For example, if we restrict sequencing viral isolates from hospitalized patients, the relationships between any variables associated with hospitalization will be distorted when compared to the general population. Thus, we would miss mutations associated with asymptomatic and symptomatic cases that did not require hospitalization, which could lead to inducing or misinterpreting the evidence for phenotype-genotype associations.^79–81^

Brito et al^82^ analyzed the spatiotemporal heterogeneity in each country’s SARS-CoV-2 genomic surveillance efforts based on metadata submitted to GISAID until May 30, 2021. These researchers estimated that when the prevalence of a rare lineage is 2%, 300 cases would need to be sequenced to detect at least 1 genome of that lineage with 95% probability. Therefore, sequencing capacity should be at least 0.5% of cases per week when incidence is >100 positive cases per 100,000 people.

Brito et al^82^ observed that countries like Denmark, which have a quick turnaround for sequencing, processing, and sharing SARS-CoV-2 genomic data (<18 days) and a high sequencing rate (>32%), observe greater lineage diversity. Many variants may be missed when sampling rates are low. However, disparities in wealth, investment in research and training, coordination, and supply chain logistics affect the ability of countries to perform genomic surveillance, especially LMICs. Therefore, efforts must be made to provide funds, training, and logistic support for researchers based in LMICs to improve their genomic surveillance capacity and public-health decision making.

## How do we classify the variants of SARS-CoV-2?

Any genome sequence that is genetically distinct from the reference can be called a variant. In practice, the SARS-CoV-2 variants represent clades that share a set of key mutations while still permitting a small amount of other sequence variation.^83,84^ Moreover, convergent evolution among geographically distant variants has been observed (Table 1).^85^ Although variants and strains are different, some researchers use these terms interchangeably (eg, Awadasseid et al,^86^ Hossein et al,^87^ and Ul-Rahman et al^88^). The term “strain” is typically associated with lineages that became sufficiently divergent to exhibit a changed phenotype.^89^

**Table 1.** Notable Variants of SARS-CoV-2 and Their Main Attributes^a^

SIG Class	WHO Class	WHO Label	Pango Lineages	CDC Lineages	First Identified	Characteristic Spike Mutations
VOC	VOC	delta (δ)	AY.1, AY.2, B.1.617.2	AY	India	T19R, K417N, L452R, T478K, P681R, D950N
VBM	VOC	alpha (α)	B.1.1.7 and Q lineages	Q	UK	HV69-, Y144-, N501Y, A570D, P681H, T716I, S982A, D1118H
VBM	VOC	beta (β)	B.1.351		South Africa	D80A, D215G, E484K, N501Y, A701V
VBM	VOC	gamma (γ)	P.1	P.1	Brazil	T20N, P26S, K417T, E484K, N501Y, T1027I
VBM	VOI	mu (μ)	B.1.621 and B.1.621.1		India	T95I, Y145N, R346K, E484K, N501Y, D614G, P681H, D950N
N/A	VOI	lambda (λ)	C.37		South America	G75V, T76I, RSYLTPG246-, L452Q, F490S, D614G, T859N
VBM	VUM	epsilon (ϵ)	B.1.427 and B.1.429		USA	S13I, W152C, L452R, D614G
VBM	VUM	eta (η)	B.1.525		UK and Nigeria	Q52R, E484K, Q677H, F888L
VBM	VUM	iota (ι)	B.1.526		USA	L5F, T95I, D253G, E484K, D614G, A701V, S477N, Q957R
VBM	VUM	kappa (κ)	B.1.617.1		India	L452R, E484Q, P681R
VBM	VUM	zeta (ζ)	P.2		Brazil	E484K
VBM	N/A	N/A	B.1.617.3		India	T19R, L452R, E484Q, P681R, D950N

Note. SIG, US government SARS-CoV-2 Interagency Group; VBM, variant being monitored; VOC, variant of concern; VOI, variant of interest; VUM, variants under monitoring; EUA, emergency use authorization.

a This table was modified and updated from the WHO website,^93^ the CDC website,^94^ Rambaut et al,^97^ and Soh et al.^167^ SIG and WHO classifications are detailed in Table 2.

In late 2020 and throughout 2021, as vaccine availability increased, information on variants began to dominate the COVID-19 response.^90–92^ The emergence of variants that might pose an increased risk to global public health prompted the WHO to characterize specific variants of interest (VOIs) and variants of concern (VOCs) to prioritize global monitoring and research.^93^ The US government SARS-CoV-2 interagency group (SIG) developed a separate variant classification scheme,^94^ which we compare to the WHO system in Table 2.

**Table 2.** Comparing the Different Categories in the WHO Variant Classification System^93^ With the System Used by the US government SARS-CoV-2 Interagency Group (SIG)^94^ ^,^^a^

SIG		WHO	
Category	Potential Attributes	Category	Working Definition
VBM	Variants for which data indicate a potential or clear impact on approved or authorized medical countermeasures or that have been associated with more severe disease or increased transmission but are no longer detected or are circulating at very low levels in the United States, and as such, do not pose a significant and imminent risk to public health in the United States	VUM	A SARS-CoV-2 variant with genetic changes that are suspected to affect virus characteristics with some indication that it may pose a future risk, but evidence of phenotypic or epidemiological impact is currently unclear, requiring enhanced monitoring and repeat assessment pending new evidence
VOI	Specific genetic markers that are predicted to affect transmission, diagnostics, therapeutics, or immune escape	VOI	With genetic changes that are predicted or known to affect virus characteristics such as transmissibility, disease severity, immune escape, diagnostic or therapeutic escape AND identified to cause significant community transmission or multiple COVID-19 clusters, in multiple countries with increasing relative prevalence alongside increasing number of cases over time, or other apparent epidemiological impacts to suggest an emerging risk to global public health
	Evidence that it is the cause of an increased proportion of cases or unique outbreak clusters		
	Limited prevalence or expansion in the United States or in other countries		
VOC	Evidence of impact on diagnostics, treatments, or vaccines	VOC	Increase in transmissibility or detrimental change in COVID-19 epidemiology OR increase in virulence or change in clinical disease presentation OR decrease in effectiveness of public health and social measures or available diagnostics, vaccines, therapeutics.
	Evidence of increased transmissibility		
	Evidence of increased disease severity		
	Evidence of increased transmissibility		
	Evidence of increased disease severity		
VOHC	Impact on medical countermeasures (MCM)		(No equivalent WHO category)
	Demonstrated failure of diagnostic test targets.		
	Evidence to suggest a significant reduction in vaccine effectiveness, a disproportionately high number of infections in vaccinated persons, or very low vaccine-induced protection against severe disease		
	Significantly reduced susceptibility to multiple EUA or approved therapeutics		
	More severe clinical disease and increased hospitalizations		

Note. VBM, variant being monitored; VOC, variant of concern; VOI, variant of interest; VUM, variants under monitoring; VOHC, variant of high consequence; EUA, emergency use authorization.

a Currently, no variants are being classified as VOI or VOHC by the CDC and SIG.

In March 2021, the WHO assigned letters of the Greek alphabet to categorize VOIs and VOCs,^93^ for simplicity and to avoid association with particular localities. These labels do not replace existing classifications by GISAID (https://gisaid.org/),^95^ Nextstrain (https://nexstrain.org/),^96^ and Pango lineages (https://cov-lineages.org/).^97^ SARS-CoV-2 variants were reviewed by Harvey et al.^98^

## Why are vaccines still not enough against COVID-19?

The speed of development and testing of COVID-19 vaccines development is one of history’s most outstanding public health achievements. Vast vaccination of eligible individuals is the best and safest way to control the pandemic.^99^ Although some SARS-CoV-2 variants show a degree of escape from protective antibodies induced by natural infection (and, to a lesser degree, after immunization), T-cell responses are retained.^100^ Furthermore, first-generation SARS-CoV-2 mRNA-based vaccines induce public antibodies (ie, antibodies with similar genetic elements and modes of recognition against a different antigen observed in multiple individuals) with robust neutralizing and potentially durable protective activity against variants such as alpha (α), beta (β), and gamma (γ).^101^

SARS-CoV-2 variants will continue to emerge,^102^ requiring close international monitoring to determine the need for vaccination boosters and or redesign.^102^ As variants emerge in areas of low vaccination, a global COVID-19 vaccination rollout is imperative. Since the vaccine rollout, new questions have arisen regarding vaccine efficacy against the transmission of different variants,^100^ the duration of protection,^103^ and the efficacy of prime-boost schedules.^99,104–106^ A demand has also arisen for studies to determine the immunological correlates of protection against COVID-19 as cases decline and prevention of severe disease gains more importance in vaccine efficacy.^107^ Meanwhile, nonpharmaceutical interventions to reduce the spread of SARS-CoV-2 and other pathogens are still warranted.^102,108,109^

## How can we bridge the knowledge gap between disease origin and transmission?

Genomic epidemiology can be a tool to study emerging infectious diseases (EIDs) in humans, but its effectiveness is maximized when it accounts for animal and environmental components. In the case of zoonosis, there is a knowledge gap between the animal and human components of EID research, and One Health can bridge this gap.

Although most human health researchers have only started focusing on coronaviruses since the emergence of SARS-CoV-2, veterinarians, virologists, and zoologists have been researching animal coronaviruses long before the COVID-19 epidemic.^110^ One Health proposes placing these realms of research (on humans and animals) in the same environmental context. The next steps in pandemic prevention science are to understand factors that create opportunities for zoonosis,^111,112^ such as entering infectious habitats such as bat caves and the use of wildlife as food and medicine.^113–117^

Deep sequencing the microbiomes and viromes of taxonomically, geographically, and temporally deep biorepository archives of putative host animals will serve as the basis of new approaches to zoonosis, risk assessment, and threat mitigation.^118–120^ Therefore, another step toward furthering the One Health approach is leveraging biorepositories in biomedical research. Although the Global Museum initiative already offers a route of international integration among museum biorepositories in a decentralized and geographically dispersed network,^121^ the link to EID research is still not fully realized.

The recent creation of the Museums and Emerging Pathogens in the Americas network (MEPA) is vital for linking biorepositories and EID research.^122^ The overarching goal of the MEPA is to leverage museum biorepositories in a global, decentralized pathogen surveillance system by expanding biodiversity infrastructure and opening communication channels that foster collaboration among biorepositories and biomedical communities.

The need for this host-based approach to genomic epidemiology is made evident by the transmissible nature of SARS-CoV-2,^123^ which has the potential to infect a range of hosts, including tigers,^124–126^ minks,^127,128^ domestic cats,^129–131^ ferrets,^132–134^ raccoon dogs,^135^ cynomolgus and rhesus macaques,^135–137^ rabbits,^138^ Egyptian fruit bats,^138,139^ Syrian hamsters,^140^ and white-tailed deer.^141–143^

## How can we track SARS-CoV-2 variants faster?

Vaccines are still effective in preventing severe outcomes against all SARS-CoV-2 variants,^100^ which are ravaging unvaccinated people.^144,145^ However, the likelihood of new mutations increases as cases rise, possibly leading to enhanced transmission, immune escape, or increased pathogenicity. This process has resulted in more transmissible variants.^146,147^

Researchers face 2 main challenges in keeping pace with SARS-CoV-2 variants: using resources at optimal capacity and lowering barriers to technology and training in genomic epidemiology across the world. On the one hand, countries with a high positivity rate, like India, are not sequencing isolates at full capacity.^148^ The United States is an even more extreme example because it has ranked low in SARS-CoV-2 sequencing despite its capacity and expertise.^149,150^ On the other hand, countries like South Africa have sequencing laboratories struggling with reagent shortages and the scarcity of trained scientists.^151^

Global efforts to strengthen pathogen sequencing capacity are still required to respond to technical, logistical, and financial challenges in resource-limited settings despite increased sequencing feasibility. Moreover, good SARS-CoV-2 sequencing performance for some LMICs (eg, Democratic Republic of the Congo, Brazil, Senegal, and Thailand) further encourages international and domestic collaboration among public health authorities, healthcare facilities, academia, and industries.^149^

Additional challenges include consistent handling of isolates as well as metadata and sequence data curation and deposition in a way that facilitates combining data sets from different laboratories. These challenges require coordinated efforts^152^ and data standards^153^ to guarantee rapid access to large volumes of raw and processed molecular data at unprecedented scales.^70^

We also need to address bioinformatics bottlenecks to respond faster to the threat of emergent diseases and to manage the fast-paced production of genomic information. Most tools are co-opted from evolutionary biology’s arsenal to study the lineages of higher taxa with exemplar approaches.^154^ Although these tools were not designed to manage big data from rapidly evolving pathogens,^154^ some have already started to respond to these demands. For example, the ultrafast sample placement on existing trees (UShER) enables the rapid placement of novel genomes into a reference tree using the parsimony optimality criterion.^155^ Thus, as phylogenetic principles underpin how we view genetic changes over time, One Health will also include the exchange of knowledge among evolutionary biologists and epidemiologists.

Phylogenetic trees are hard to compute and interpret. The need to consult professional phylogeneticists is made plain by the number of prominent papers that did not adhere to the standards of phylogenetics and failed to identify the fundamental hosts of coronaviruses.^156^ Moreover, a good phylogenetic analysis requires many elements: careful choice of the collected taxa, sequence, and or phenotypic data; method and quality control of sequence data and alignment; evaluation of substitution and indel models; treatment of partitions; tree-search protocol; measures of fit or confidence; and strategies for character coding and optimization.^49,156,157^ Moreover, results may vary with parameterization.^158^ These are only a few of the difficult decisions that go way beyond the level of sophistication of any software manuals and automated systems.^156,159^

## Are trees mapped to globes always needed?

In many cases, such as the initial spread of H5N1 influenza, trees and Supramaps were very useful to understand the geographic spread of the pathogen, its multiple geographically and mutationally distinct patterns of zoonosis,^10^ and drug resistance.^14^ However, due to occlusion, Supramaps were not suitable for the visualization of cosmopolitan diseases, such as strains of *Salmonella* (eg, Hoffman et al^3^), seasonal influenza (eg, H3N2), pandemic influenza (H1N1-2009),^16^ and SARS-CoV-2. In response, researchers have worked on alternative visualization tools, including pointmaps and route maps^13,160^ and eventually moved beyond the need for mapping trees to globes with Strainhub.^161^

**Fig. 5.**
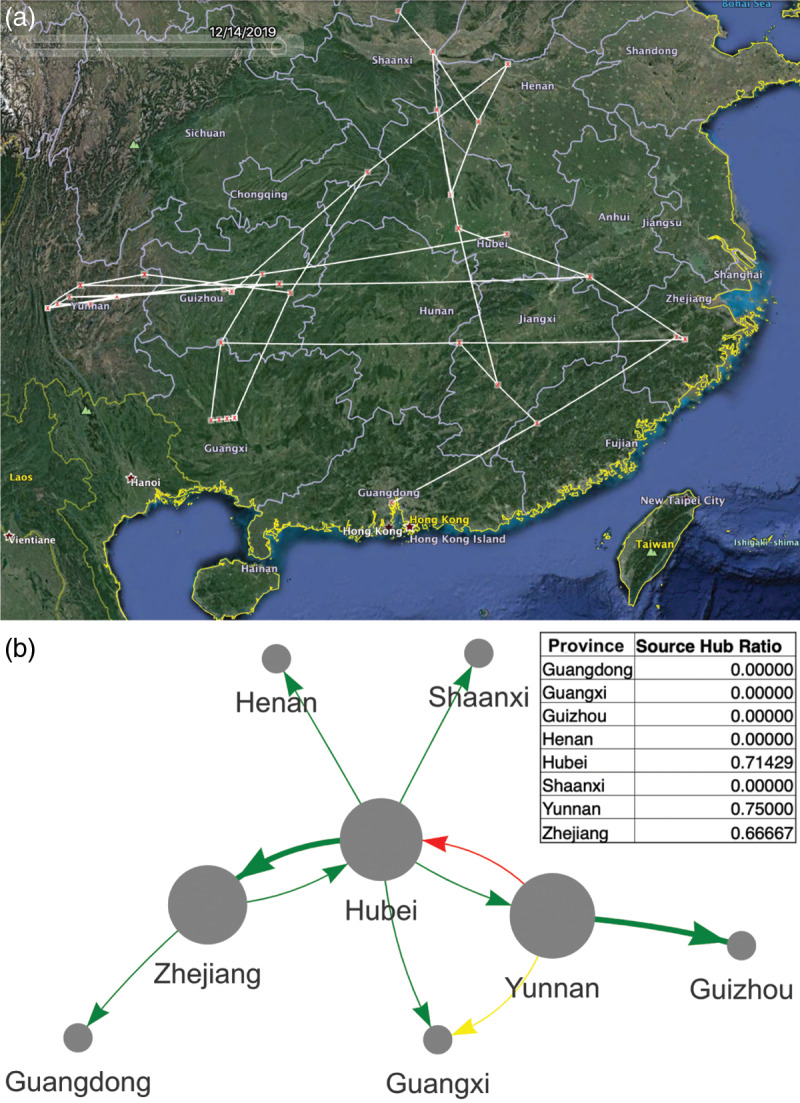
Comparison between Supramap and Strainhub visualizations. (a) Supramap phylogenetic visualization of bat-hosted and pangolin-hosted coronaviruses that share recent ancestry (2005–2019) with human-hosted SARS-CoV-2. The underlying data are genomic sequences, temporal and geographic metadata. (b) Strainhub visualization of the same data plus host metadata in a network using arbitrary space. Arrow colors correspond to different types of transmission (red = bat to human, green = bat to bat, yellow = bat to pangolin). The size of the circle represents the source hub ratio (SHR). SHR is the number of transitions originating from a node as a fraction of the total number of transitions related to that node. A node scoring SHR close to 1 indicates a source (eg, Hubei, Yunnan, and Zhejiang), SHR close to 0.5 a hub and SHR close to 0 a sink for the pathogen. The thickness of the line represents a higher frequency of viral transmission (eg, Hubei to Zhejiang).

Unlike Supramap, Strainhub is less computationally demanding. It can be executed from a web browser; it does not depend on closed source software (Google Earth), and geographical data are optional (Fig. 5). Moreover, Strainhub can be used to test hypotheses on the relative importance of hosts or places in disease spread. Future efforts for Strainhub will focus on usability, interoperability, visual clarity, and quantification of the relative importance of hosts or places in the spread of disease to better understand zoonosis.

## How do we prepare for the next pandemic?

The COVID-19 pandemic has illustrated how unprepared our interconnected global society is for zoonotic disease. For the next pandemic, 2 frontiers of investigation are interesting for genomic epidemiology as a tool to survey microbes of pandemic potential to predict, prevent, or respond faster to the emergence of new disease.

First, we must survey the natural diversity of coronaviruses and other microbes of pandemic potential present within animals.^120^ Second, we must develop the science of pandemic prevention by moving from tracking pandemics that are occurring to predicting outbreaks. For example, combining artificial intelligence with genomic epidemiology can lead constructing a “viral forecast“ to inform decisions about viruses with pandemic potential.^162^ Moreover, we have proposed a novel mathematical modeling framework based on agent-based modeling to predict pathogen patch dynamics underlying zoonosis.^163^

## Final remarks

The COVID-19 pandemic, while ongoing, has caused 4,863,818 deaths worldwide as of October 14, 2021,^164^ and it has surpassed the US death toll from the 1918–1919 H1N1 pandemic, which was ∼675,000. As SARS-CoV-2 becomes endemic, we must remember that it is not as lethal as other pathogens such as H5N1 influenza or Nipah virus. In its last 100 years of existence, smallpox killed 300 million people, and *Variola major* (the major variant of smallpox) killed 30% of these patients.^165^

A novel pathogen at 30% mortality infecting 50% of the US population (166.7 million) would have resulted in 50 million deaths. MERS-CoV, henipaviruses, and hantavirus all have high mortality (>30%) and virulence with no approved vaccines or antivirals available. The 2018 Nipah outbreak had a 91% case-fatality rate, claiming 21 lives.^166^ We must heed the warning that pathogens with more severe disease phenotypes than SARS-CoV-2 could resultin a far more devastating pandemic.

## Supplementary material

For supplementary material accompanying this paper visit https://doi.org/10.1017/ash.2021.222. click here to view supplementary material

## References

[1] Janies DA. Phylogenetic concepts and tools applied to epidemiologic investigations of infectious diseases. *Microbiol Spectr* 2019;7. doi: 10.1128/microbiolspec.AME-0006-2018. Accessed November 10, 2021. PMC10956736 31325287

[2] Janies DA , Pomeroy LW , Aaronson JM , et al. Analysis and visualization of H7 influenza using genomic, evolutionary and geographic information in a modular web service. Cladistics 2012;28:483–488. 32313365 10.1111/j.1096-0031.2012.00401.x PMC7162197

[3] Hoffmann M , Luo Y , Monday SR , et al. Tracing origins of the *Salmonella* Bareilly strain causing a foodborne outbreak in the United States. J Infect Dis 2016;213:502–508. 25995194 10.1093/infdis/jiv297

[4] Ezeoke I , Galac MR , Lin Y , et al. Tracking a serial killer: integrating phylogenetic relationships, epidemiology, and geography for two invasive meningococcal disease outbreaks. PLoS One 2018;13: e0202615. 30485280 10.1371/journal.pone.0202615 PMC6261407

[5] Allard MW , Strain E , Melka D , et al. Practical value of food pathogen traceability through building a whole-genome sequencing network and database. J Clin Microbiol 2016;54: 1975–1983. 27008877 10.1128/JCM.00081-16 PMC4963501

[6] Marra MA , Jones SJM , Astell CR , et al. The genome sequence of the SARS-associated coronavirus. Science 2003;300:1399–1404. 12730501 10.1126/science.1085953

[7] Rota PA , Oberste MS , Monroe SS , et al. Characterization of a novel coronavirus associated with severe acute respiratory syndrome. Science 2003;300:1394–1399. 12730500 10.1126/science.1085952

[8] Boulos MNK. Descriptive review of geographic mapping of severe acute respiratory syndrome (SARS) on the Internet. Int J Health Geogr 2004;3:2. 14748926 10.1186/1476-072X-3-2 PMC343293

[9] Janies DA , Habib F , Alexandrov B , Hill A , Pol D. Evolution of genomes, host shifts and the geographic spread of SARS-CoV and related coronaviruses. Cladistics 2008;24:111–130. 32313363 10.1111/j.1096-0031.2008.00199.x PMC7162247

[10] Janies DA , Hill AW , Guralnick R , Habib F , Waltari E , Wheeler WC. Genomic analysis and geographic visualization of the spread of avian influenza (H5N1). Syst Biol 2007;56:321–329. 17464886 10.1080/10635150701266848

[11] Janies DA , Treseder T , Alexandrov B , et al. The Supramap project: linking pathogen genomes with geography to fight emergent infectious diseases. Cladistics 2011;27:61–66. 32313364 10.1111/j.1096-0031.2010.00314.x PMC7162175

[12] Studer J , Janies DA. Global spread and evolution of viral haemorrhagic septicaemia virus. J Fish Dis 2011;34:741–747. 21916899 10.1111/j.1365-2761.2011.01290.x

[13] Janies DA , Voronkin IO , Studer J , et al. Selection for resistance to oseltamivir in seasonal and pandemic H1N1 influenza and widespread co-circulation of the lineages. Int J Health Geogr 2010;9:13. 20181276 10.1186/1476-072X-9-13 PMC2882220

[14] Hill AW , Guralnick RP , Wilson MJC , Habib F , Janies D. Evolution of drug resistance in multiple distinct lineages of H5N1 avian influenza. Infect Genet Evol 2009;9:169–178. 19022400 10.1016/j.meegid.2008.10.006

[15] Testimony of Daniel A. Janies, PhD. Local challenges of global proportions: evaluating role, preparedness for, and surveillance for pandemic onfluenza: Hearing before the committee on homeland security and government affairs, United States senate, 1 Sess. (2007). US government website. https://www.govinfo.gov/content/pkg/CHRG-110shrg38846/html/CHRG-110shrg38846.htm. Accessed October 7, 2021.

[16] Janies DA , Voronkin IO , Das M , Hardman J , Treseder TW , Studer J. Genome informatics of influenza A: from data sharing to shared analytical capabilities. Anim Health Res Rev 2010;11:73–79. 20591214 10.1017/S1466252310000083

[17] Ruan YJ , Wei CL , Ee AL , et al. Comparative full-length genome sequence analysis of 14 SARS coronavirus isolates and common mutations associated with putative origins of infection. Lancet 2003;361:1779–1785. 12781537 10.1016/S0140-6736(03)13414-9 PMC7140172

[18] Zhao G-P. SARS molecular epidemiology: a Chinese fairy tale of controlling an emerging zoonotic disease in the genomics era. Philos Trans R Soc Lond B Biol Sci 2007;362:1063–1081. 17327210 10.1098/rstb.2007.2034 PMC2435571

[19] Reuter JA , Spacek DV , Snyder MP. High-throughput sequencing technologies. Mol Cell 2015;58:586–597. 26000844 10.1016/j.molcel.2015.05.004 PMC4494749

[20] Pérez-Losada M , Arenas M , Galán JC , et al. High-throughput sequencing (HTS) for the analysis of viral populations. Infect Genet Evol 2020;80:104208. 32001386 10.1016/j.meegid.2020.104208

[21] The cost of sequencing a human genome. National Human Genome Research Institute website. https://www.genome.gov/about-genomics/fact-sheets/Sequencing-Human-Genome-cost. Accessed September 6, 2021.

[22] Woo PCY , Lau SKP , Lam CSF , et al. Discovery of a novel bottlenose dolphin coronavirus reveals a distinct species of marine mammal coronavirus in gamma coronavirus. J Virol 2014;88:1318–1331. 24227844 10.1128/JVI.02351-13 PMC3911666

[23] Durães-Carvalho R , Caserta LC , Barnabé ACS , et al. Coronaviruses detected in Brazilian wild birds reveal close evolutionary relationships with beta- and deltacoronaviruses isolated from mammals. J Mol Evol 2015;81:21–23. 26250156 10.1007/s00239-015-9693-9 PMC7079945

[24] Woo PCY , Lau SKP , Lam CSF , et al. Discovery of seven novel mammalian and avian coronaviruses in the genus deltacoronavirus supports bat coronaviruses as the gene source of alphacoronavirus and betacoronavirus and avian coronaviruses as the gene source of gammacoronavirus and deltacoronavirus. J Virol 2012;86:3995–4008. 22278237 10.1128/JVI.06540-11 PMC3302495

[25] Woo PCY , Huang Y , Lau SKP , Yuen K-Y. Coronavirus genomics and bioinformatics analysis. Viruses. 2010;2:1804–1820. 21994708 10.3390/v2081803 PMC3185738

[26] Irigoyen N , Firth AE , Jones JD , Chung BY-W , Siddell SG , Brierley I. High-resolution analysis of coronavirus gene expression by RNA sequencing and ribosome profiling. PLoS Pathog 2016;12:e1005473. 26919232 10.1371/journal.ppat.1005473 PMC4769073

[27] Coronavirinae. ViralZone website. https://viralzone.expasy.org/785?outline=all_by_species. Accessed September 8, 2021.

[28] Li BX , Ge JW , Li YJ. Porcine aminopeptidase N is a functional receptor for the PEDV coronavirus. Virology. 2007;365:166–172. 17467767 10.1016/j.virol.2007.03.031 PMC7103304

[29] Boileau MJ , Kapil S. Bovine coronavirus associated syndromes. Vet Clin N Am Food Anim Pract 2010;26:123–146. 10.1016/j.cvfa.2009.10.003 PMC7125561 20117547

[30] Mandelik R , Sarvas M , Jackova A , Salamunova S , Novotny J , Vilcek S. First outbreak with chimeric swine enteric coronavirus (SeCoV) on pig farms in Slovakia—lessons to learn. Acta Vet Hung 2018;66:488–492. 30264613 10.1556/004.2018.043

[31] Su S , Wong G , Shi W , Liu J , Lai ACK , Zhou J , et al. Epidemiology, genetic recombination, and pathogenesis of coronaviruses. Trends Microbiol 2016;24:490–502. 27012512 10.1016/j.tim.2016.03.003 PMC7125511

[32] Lai MMC , Cavanagh D. The molecular biology of coronaviruses. In Advances in Virus Research. New York: Elsevier; 1997: 1–100. 10.1016/S0065-3527(08)60286-9 PMC7130985 9233431

[33] Human coronavirus types. Centers for Disease Control and Prevention website. https://www.cdc.gov/coronavirus/types.html. Published March 17, 2021. Accessed September 7, 2021.

[34] Zhang XM , Herbst W , Kousoulas KG , Storz J. Biological and genetic characterization of a hemagglutinating coronavirus isolated from a diarrhoeic child. J Med Virol 1994;44:152–161. 7852955 10.1002/jmv.1890440207 PMC7166597

[35] Zhong NS , Zheng BJ , Li YM , et al. Epidemiology and cause of severe acute respiratory syndrome (SARS) in Guangdong, People’s Republic of China, in February 2003. Lancet 2003;362:1353–1358. 14585636 10.1016/S0140-6736(03)14630-2 PMC7112415

[36] Ksiazek TG , Erdman D , Goldsmith CS , et al. A novel coronavirus associated with severe acute respiratory syndrome. N Engl J Med 2003;348:1953–1966. 12690092 10.1056/NEJMoa030781

[37] Zumla A , Hui DS , Perlman S. Middle East respiratory syndrome. Lancet 2015;386:995–1007. 26049252 10.1016/S0140-6736(15)60454-8 PMC4721578

[38] World Health Organization director-general’s opening remarks at the media briefing on COVID-19— 11 March 2020. World Health Organization website. https://www.who.int/dg/speeches/detail/who-director-general-s-opening-remarks-at-the-media-briefing-on-covid-19---11-march-2020. Accessed September 7, 2021.

[39] Pekar J , Worobey M , Moshiri N , Scheffler K , Wertheim JO. Timing the SARS-CoV-2 index case in Hubei province. Science 2021;372:412–417. 33737402 10.1126/science.abf8003 PMC8139421

[40] Coronavirus (COVID-19) dashboard. World Health Organization website. https://covid19.who.int. Accessed September 18, 2021.

[41] Yuen K-S , Ye Z-W , Fung S-Y , Chan C-P , Jin D-Y. SARS-CoV-2 and COVID-19: the most important research questions. Cell Biosci 2020;10:40. 32190290 10.1186/s13578-020-00404-4 PMC7074995

[42] Andersen KG , Rambaut A , Lipkin WI , Holmes EC , Garry RF. The proximal origin of SARS-CoV-2. Nat Med 2020;26:450–452. 32284615 10.1038/s41591-020-0820-9 PMC7095063

[43] Liu S-L , Saif LJ , Weiss SR , Su L. No credible evidence supporting claims of the laboratory engineering of SARS-CoV-2. Emerg Microbes Infect 2020;9:505–507. 32102621 10.1080/22221751.2020.1733440 PMC7054935

[44] Holmes EC , Goldstein SA , Rasmussen AL , et al. The origins of SARS-CoV-2: a critical review. *Cell* 2021. doi: 10.1016/j.cell.2021.08.017. PMC8373617 34480864

[45] Wang N , Li S-Y , Yang X-L , et al. Serological evidence of bat SARS-related coronavirus infection in humans, China. Virol Sin 2018;33:104–107. 29500691 10.1007/s12250-018-0012-7 PMC6178078

[46] Zhang X , Hasoksuz M , Spiro D , et al. Quasi-species of bovine enteric and respiratory coronaviruses based on complete genome sequences and genetic changes after tissue culture adaptation. Virology 2007;363:1–10. 17434558 10.1016/j.virol.2007.03.018 PMC7103286

[47] Rasmussen AL. On the origins of SARS-CoV-2. *Nat Med* 2021. doi: 10.1038/s41591-020-01205-5. 33442004

[48] Shi Z-L. Origins of SARS-CoV-2: focusing on science. Infect Dis Immun 2021;1:3–4. 10.1097/ID9.0000000000000008 PMC8057312 38630114

[49] Machado DJ , Scott R , Guirales S , Janies DA. Fundamental evolution of all including three deadly lineages descendent from Chiroptera-hosted coronaviruses: SARS-CoV, MERS-CoV, and SARS-CoV-2. *Cladistics* 2021. doi: 10.1111/cla.12454. PMC8239696 34570933

[50] Zhao S , Zhuang Z , Cao P , et al. Quantifying the association between domestic travel and the exportation of novel coronavirus (2019-nCoV) cases from Wuhan, China in 2020: a correlational analysis. *J Travel Med* 2020;27. doi: 10.1093/jtm/taaa022. PMC7107546 32080723

[51] Li W , Shi Z , Yu M , et al. Bats are natural reservoirs of SARS-like coronaviruses. Science 2005;310:676–679. 16195424 10.1126/science.1118391

[52] Lytras S , Hughes J , Martin D , et al. Exploring the natural origins of SARS-CoV-2 in the light of recombination. *bioRxiv* 2021. doi: 10.1101/2021.01.22.427830. PMC8882382 35137080

[53] Lytras S , Xia W , Hughes J , Jiang X , Robertson DL. The animal origin of SARS-CoV-2. Science 2021;373:968–970. 34404734 10.1126/science.abh0117

[54] Lam TT-Y , Jia N , Zhang Y-W , et al. Identifying SARS-CoV-2-related coronaviruses in Malayan pangolins. Nature 2020;583:282–285. 32218527 10.1038/s41586-020-2169-0

[55] Xiao K , Zhai J , Feng Y , et al. Isolation of SARS-CoV-2-related coronavirus from Malayan pangolins. Nature 2020;583:286–289. 32380510 10.1038/s41586-020-2313-x

[56] Zhang T , Wu Q , Zhang Z. Probable pangolin origin of SARS-CoV-2 associated with the COVID-19 outbreak. Curr Biol 2020;30:1346–1351.e2. 32197085 10.1016/j.cub.2020.03.022 PMC7156161

[57] Liu P , Jiang J-Z , Wan X-F , et al. Are pangolins the intermediate host of the 2019 novel coronavirus (SARS-CoV-2)? PLoS Pathog 2020;16:e1008421. 32407364 10.1371/journal.ppat.1008421 PMC7224457

[58] GISAID—Initiative. Global Initiative on Sharing All Influenza Data website. https://www.gisaid.org. Accessed September 18, 2021.

[59] GISAID—Initiative. EpiCov website. https://www.epicov.org/. Accessed September 18, 2021.

[60] Wu F , Zhao S , Yu B , et al. Author correction: a new coronavirus associated with human respiratory disease in China. Nature 2020;580:E7. 32296181 10.1038/s41586-020-2202-3 PMC7608129

[61] Sequencing of SARS-CoV-2. European Centre for Disease Control and Prevention website. https://www.ecdc.europa.eu/sites/default/files/documents/sequencing-of-SARS-CoV-2.pdf Accessed September 18, 2021.

[62] Genomic sequencing of SARS-CoV-2: a guide to implementation for maximum impact on public health. World Health Organization website. https://www.who.int/publications/i/item/9789240018440. Published January 2021. Accessed September 18, 2021.

[63] Truelove S , Smith CP , Qin M , et al. Projected resurgence of COVID-19 in the United States in July–December 2021 resulting from the increased transmissibility of the Delta variant and faltering vaccination. *medRxiv* 2021. doi: 10.1101/2021.08.28.21262748. PMC9232215 35726851

[64] Lau BT , Pavlichin D , Hooker AC , et al. Profiling SARS-CoV-2 mutation fingerprints that range from the viral pangenome to individual infection quasispecies. Genome Med 2021;13:62. 33875001 10.1186/s13073-021-00882-2 PMC8054698

[65] Hamza M , Ali A , Khan S , et al. nCOV-19 peptides mass fingerprinting identification, binding, and blocking of inhibitors flavonoids and anthraquinone of and hydroxychloroquine. J Biomol Struct Dyn 2021;39:4089–4099. 32567487 10.1080/07391102.2020.1778534 PMC7332867

[66] Hariton E , Locascio JJ. Randomised controlled trials—the gold standard for effectiveness research: Study design: randomised controlled trials. BJOG 2018;125:1716. 29916205 10.1111/1471-0528.15199 PMC6235704

[67] Boulware DR , Pullen MF , Bangdiwala AS , et al. A randomized trial of hydroxychloroquine as postexposure prophylaxis for COVID-19. N Engl J Med. 2020;383:517–525. 32492293 10.1056/NEJMoa2016638 PMC7289276

[68] Siemieniuk RA , Bartoszko JJ , Ge L , et al. Drug treatments for COVID-19: living systematic review and network meta-analysis. BMJ 2020;370:m2980. 32732190 10.1136/bmj.m2980 PMC7390912

[69] COVID-19 mRNA vaccine production. National Human Genome Research Institute website. https://www.genome.gov/about-genomics/fact-sheets/COVID-19-mRNA-Vaccine-Production. Accessed October 12, 2021.

[70] Chiara M , D’Erchia AM , Gissi C , et al. Next-generation sequencing of SARS-CoV-2 genomes: challenges, applications and opportunities. Brief Bioinform 2021;22:616–630. 33279989 10.1093/bib/bbaa297 PMC7799330

[71] Shaman J , Galanti M. Will SARS-CoV-2 become endemic? Science 2020;370:527–529. 33055131 10.1126/science.abe5960

[72] Nakanishi N , Yoshio I. The novel coronavirus pandemic and the state of the epidemic in Kobe, Japan. J Disaster Res 2021;16:84–87.

[73] Phillips N. The coronavirus is here to stay—here’s what that means. Nature 2021;590:382–384. 33594289 10.1038/d41586-021-00396-2

[74] Zhou D , Dejnirattisai W , Supasa P , et al. Evidence of escape of SARS-CoV-2 variant B.1.351 from natural and vaccine-induced sera. Cell 2021;184:2348–2361.e6. 33730597 10.1016/j.cell.2021.02.037 PMC7901269

[75] Kustin T , Harel N , Finkel U , et al. Evidence for increased breakthrough rates of SARS-CoV-2 variants of concern in BNT162b2-mRNA-vaccinated individuals. Nat Med 2021;27:1379–1384. 34127854 10.1038/s41591-021-01413-7 PMC8363499

[76] Farinholt T , Doddapaneni H , Qin X , et al. Transmission event of SARS-CoV-2 delta variant reveals multiple vaccine breakthrough infections. BMC Med 2021;19:255. 34593004 10.1186/s12916-021-02103-4 PMC8483940

[77] Giovanetti M , Benedetti F , Campisi G , et al. Evolution patterns of SARS-CoV-2: snapshot on its genome variants. Biochem Biophys Res Commun 2021;538:88–91. 33199021 10.1016/j.bbrc.2020.10.102 PMC7836704

[78] To KK-W , Sridhar S , Chiu KH-Y , et al. Lessons learned 1 year after SARS-CoV-2 emergence leading to COVID-19 pandemic. Emerg Microbes Infect 2021;10:507–535. 33666147 10.1080/22221751.2021.1898291 PMC8006950

[79] Munafò MR , Tilling K , Taylor AE , Evans DM , Davey Smith G. Collider scope: when selection bias can substantially influence observed associations. Int J Epidemiol 2018;47:226–235. 29040562 10.1093/ije/dyx206 PMC5837306

[80] Hernán MA. Invited commentary: selection bias without colliders. Am J Epidemiol 2017;185:1048–1050. 28535177 10.1093/aje/kwx077 PMC6664806

[81] Tattan-Birch H , Marsden J , West R , Gage SH. Assessing and addressing collider bias in addiction research: the curious case of smoking and COVID-19. Addiction 2021;116:982–984. 33226690 10.1111/add.15348 PMC7753816

[82] Brito AF , Semenova E , Dudas G , et al. Global disparities in SARS-CoV-2 genomic surveillance. *medRxiv* 2021. doi: 10.1101/2021.08.21.21262393. PMC9667854 36385137

[83] Lauring AS , Hodcroft EB. Genetic variants of SARS-CoV-2-what do they mean? JAMA 2021;325:529–531. 33404586 10.1001/jama.2020.27124

[84] Tegally H , Wilkinson E , Giovanetti M , et al. Emergence and rapid spread of a new severe acute respiratory syndrome-related coronavirus 2 (SARS-CoV-2) lineage with multiple spike mutations in South Africa. *medRxiv* 2020. doi: 10.1101/2020.12.21.20248640.

[85] Ford CT , Scott R , Machado DJ , Janies D. Sequencing data of North American SARS-CoV-2 isolates shows widespread complex variants. *medRxiv* 2021. doi: 10.1101/2021.01.27.21250648.

[86] Awadasseid A , Wu Y , Tanaka Y , Zhang W. Current advances in the development of SARS-CoV-2 vaccines. Int J Biol Sci 2021;17:8–19. 33390829 10.7150/ijbs.52569 PMC7757035

[87] Hossain MK , Hassanzadeganroudsari M , Apostolopoulos V. The emergence of new strains of SARS-CoV-2. What does it mean for COVID-19 vaccines? Expert Rev Vaccines 2021;20:635–638. 33896316 10.1080/14760584.2021.1915140 PMC8074646

[88] Ul-Rahman A , Shabbir MAB , Aziz MW , et al. A comparative phylogenomic analysis of SARS-CoV-2 strains reported from non-human mammalian species and environmental samples. Mol Biol Rep 2020;47:9207–9217. 33104993 10.1007/s11033-020-05879-5 PMC7586201

[89] Kuhn JH , Bao Y , Bavari S , et al. Virus nomenclature below the species level: a standardized nomenclature for natural variants of viruses assigned to the family *Filoviridae* . Arch Virol 2013;158:301–311. 23001720 10.1007/s00705-012-1454-0 PMC3535543

[90] Parums D. Editorial: revised World Health Organization (WHO) terminology for variants of concern and variants of interest of SARS-CoV-2. Med Sci Monit 2021;27:e933622. 34149046 10.12659/MSM.933622 PMC8230247

[91] Konings F , Perkins MD , Kuhn JH , et al. SARS-CoV-2 variants of interest and concern naming scheme conducive for global discourse. Nat Microbiol 2021;6:821–823. 34108654 10.1038/s41564-021-00932-w

[92] Janik E , Niemcewicz M , Podogrocki M , Majsterek I , Bijak M. The emerging concern and interest SARS-CoV-2 variants. *Pathogens* 2021;10. doi: 10.3390/pathogens10060633. PMC8224338 34064143

[93] Tracking SARS-CoV-2 variants. World Health Organization website. https://www.who.int/activities/tracking-SARS-CoV-2-variants. Accessed September 20, 2021.

[94] SARS-CoV-2 variant classifications and definitions. Centers for Disease Control and Prevention website. https://www.cdc.gov/coronavirus/2019-ncov/variants/variant-info.html. Published 2021. Accessed September 20, 2021.

[95] Shu Y , McCauley J. GISAID: Global initiative on sharing all influenza data—from vision to reality. *Euro Surveill* 2017;22. doi: 10.2807/1560-7917.ES.2017.22.13.30494. PMC5388101 28382917

[96] Hadfield J , Megill C , Bell SM , et al. Nextstrain: real-time tracking of pathogen evolution. Bioinformatics 2018;34:4121–4123. 29790939 10.1093/bioinformatics/bty407 PMC6247931

[97] Rambaut A , Holmes EC , O’Toole Á , et al. A dynamic nomenclature proposal for SARS-CoV-2 lineages to assist genomic epidemiology. Nat Microbiol 2020;5:1403–1407. 32669681 10.1038/s41564-020-0770-5 PMC7610519

[98] Harvey WT , Carabelli AM , Jackson B , et al. SARS-CoV-2 variants, spike mutations and immune escape. Nat Rev Microbiol 2021;19:409–424. 34075212 10.1038/s41579-021-00573-0 PMC8167834

[99] Flanagan KL , MacIntyre CR , McIntyre PB , Nelson MR. SARS-CoV-2 vaccines: where are we now? *J Allergy Clin Immunol Pract* 2021. doi: 10.1016/j.jaip.2021.07.016. PMC8363243 34400116

[100] Cevik M , Grubaugh ND , Iwasaki A , Openshaw P. COVID-19 vaccines: keeping pace with SARS-CoV-2 variants. *Cell* 2021. doi: 10.1016/j.cell.2021.09.010. PMC8445744 34534444

[101] Schmitz AJ , Turner JS , Liu Z , et al. A vaccine-induced public antibody protects against SARS-CoV-2 and emerging variants. Immunity 2021;54:2159–2166. 34464596 10.1016/j.immuni.2021.08.013 PMC8367776

[102] Boehm E , Kronig I , Neher RA , et al. Novel SARS-CoV-2 variants: the pandemics within the pandemic. Clin Microbiol Infect 2021;27:1109–1117. 34015535 10.1016/j.cmi.2021.05.022 PMC8127517

[103] Farooqi T , Malik JA , Mulla AH , et al. An overview of SARS-COV-2 epidemiology, mutant variants, vaccines, and management strategies. *J Infect Public Health* 2021. doi: 10.1016/j.jiph.2021.08.014. PMC8366110 34429257

[104] Krause PR , Gruber MF. Emergency use authorization of COVID vaccines—safety and efficacy follow-up considerations. N Engl J Med 2020;383:e107. 33064383 10.1056/NEJMp2031373

[105] Pascual-Iglesias A , Canton J , Ortega-Prieto AM , Jimenez-Guardeño JM , Regla-Nava JA. An overview of vaccines against SARS-CoV-2 in the COVID-19 pandemic era. *Pathogens* 2021;10. doi: 10.3390/pathogens10081030. PMC8402174 34451494

[106] Chen Y , Zhu L , Huang W , et al. Potent RBD-specific neutralizing rabbit monoclonal antibodies recognize emerging SARS-CoV-2 variants elicited by DNA prime-protein boost vaccination. Emerg Microbes Infect 2021;10:1390–1403. 34120577 10.1080/22221751.2021.1942227 PMC8274519

[107] Hodgson SH , Mansatta K , Mallett G , Harris V , Emary KRW , Pollard AJ. What defines an efficacious COVID-19 vaccine? A review of the challenges assessing the clinical efficacy of vaccines against SARS-CoV-2. Lancet Infect Dis 2021;21:e26–e35. 33125914 10.1016/S1473-3099(20)30773-8 PMC7837315

[108] Zhao T , Hu C , Ayaz Ahmed M , Cheng C , Chen Y , Sun C. Warnings regarding the potential coronavirus disease 2019 (COVID-19) transmission risk: vaccination is not enough. *Infect Control Hosp Epidemiol* 2021;2. doi: 10.1016/j.xinn.2021.100116. PMC7948099 33563345

[109] Lanzavecchia S , Beyer KJ , Evina Bolo S. Vaccination is not enough: understanding the increase in cases of COVID-19 in Chile despite a high vaccination rate. Epidemiologia 2021;2:377–390. 10.3390/epidemiologia2030028 PMC9620875 36417232

[110] Poudel U , Subedi D , Pantha S , Dhakal S. Animal coronaviruses and coronavirus disease 2019: lesson for One Health approach. Open Vet J 2020;10:239–251. 33282694 10.4314/ovj.v10i3.1 PMC7703617

[111] Semenza JC , Menne B. Climate change and infectious diseases in Europe. Lancet Infect Dis 2009;9:365–375. 19467476 10.1016/S1473-3099(09)70104-5

[112] Bartlow AW , Manore C , Xu C , et al. Forecasting zoonotic infectious disease response to climate change: mosquito vectors and a changing environment. *Vet Sci China* 2019;6. doi: 10.3390/vetsci6020040. PMC6632117 31064099

[113] Mersha C , Tewodros F. One Health , one medicine, one world: co-joint of animal and human medicine with perspectives, a review. *Veterinary World* 2012. doi: 10.5455/vetworld.2012.238-243

[114] Sánchez-Vizcaíno JM. One world, One Health, one virology. *Vet Microbiol* 2013. doi: 10.1016/j.vetmic.2013.02.018. 23517762

[115] Reeve-Johnson L. One Health and a world of opportunity. *Veterinary Record* 2015. doi: 10.1136/vr.h2117. 25908758

[116] Mwangi W , de Figueiredo P , Criscitiello MF. One Health: addressing global challenges at the nexus of human, animal, and environmental Health. PLoS Pathog 2016;12:e1005731. 27631500 10.1371/journal.ppat.1005731 PMC5025119

[117] Kelly TR , Karesh WB , Johnson CK , et al. One Health proof of concept: bringing a transdisciplinary approach to surveillance for zoonotic viruses at the human–wild animal interface. Prev Vet Med 2017;137:112–118. 28034593 10.1016/j.prevetmed.2016.11.023 PMC7132382

[118] Colella JP , Stephens RB , Campbell ML , Kohli BA , Parsons DJ , Mclean BS. The open-specimen movement. Bioscience 2021;71:405–414.

[119] Cook JA , Arai S , Armién B , et al. Integrating biodiversity infrastructure into pathogen discovery and mitigation of emerging infectious diseases. Bioscience 2020;70:531–534. 32665736 10.1093/biosci/biaa064 PMC7340541

[120] Thompson CW , Phelps KL , Allard MW , et al. Preserve a voucher specimen! The critical need for integrating natural history collections in infectious disease studies. *MBio* 2021;12. doi: 10.1128/mBio.02698-20. PMC7844540 33436435

[121] Bakker FT , Antonelli A , Clarke JA , et al. The Global Museum: natural history collections and the future of evolutionary science and public education. Peer J 2020;8:e8225. 32025365 10.7717/peerj.8225 PMC6993751

[122] Colella JP , Bates J , Burneo SF , et al. Leveraging natural history biorepositories as a global, decentralized, pathogen surveillance network. PLoS Pathog 2021;17:e1009583. 34081744 10.1371/journal.ppat.1009583 PMC8174688

[123] Conceicao C , Thakur N , Human S , et al. The SARS-CoV-2 spike protein has a broad tropism for mammalian ACE2 proteins. PLoS Biol 2020;18:e3001016. 33347434 10.1371/journal.pbio.3001016 PMC7751883

[124] Wang L , Mitchell PK , Calle PP , et al. Complete genome sequence of SARS-CoV-2 in a tiger from a US zoological collection. *Microbiol Resour Announc* 2020;9. doi: 10.1128/MRA.00468-20. PMC7256270 32467283

[125] McAloose D , Laverack M , Wang L , et al. From people to: natural SARS-CoV-2 infection in tigers and lions at the Bronx Zoo. *MBio* 2020;11. doi: 10.1128/mBio.02220-20. PMC7554670 33051368

[126] Bartlett SL , Diel DG , Wang L , et al. SARS-CoV-2 infection and longitudinal fecal screening in Malayan tigers (*Panthera tigris jacksoni*), Amur tigers (*Panthera tigris altaica*), and African lions (*Panthera leo krugeri*) at the Bronx Zoo, New York, USA. J Zoo Wildl Med 2021;51:733–744. 33480553 10.1638/2020-0171

[127] Oreshkova N , Molenaar RJ , Vreman S , et al. SARS-CoV-2 infection in farmed minks, the Netherlands, April and May 2020. *Euro Surveill* 2020;25. doi: 10.2807/1560-7917.ES.2020.25.23.2001005. PMC7403642 32553059

[128] Hammer AS , Quaade ML , Rasmussen TB , et al. SARS-CoV-2 transmission between mink (*Neovison vison*) and humans, Denmark. Emerg Infect Dis 2021;27:547–551. 33207152 10.3201/eid2702.203794 PMC7853580

[129] Halfmann PJ , Hatta M , Chiba S , et al. Transmission of SARS-CoV-2 in domestic cats. N Engl J Med 2020;383:592–594. 32402157 10.1056/NEJMc2013400 PMC9678187

[130] Gaudreault NN , Trujillo JD , Carossino M , et al. SARS-CoV-2 infection, disease and transmission in domestic cats. Emerg Microbes Infect 2020;9:2322–2332. 33028154 10.1080/22221751.2020.1833687 PMC7594869

[131] Braun KM , Moreno GK , Halfmann PJ , et al. Transmission of SARS-CoV-2 in domestic cats imposes a narrow bottleneck. PLoS Pathog 2021;17:e1009373. 33635912 10.1371/journal.ppat.1009373 PMC7946358

[132] Liu H-L , Yeh I-J , Phan NN , et al. Gene signatures of SARS-CoV/SARS-CoV-2–infected ferret lungs in short- and long-term models. Infect Genet Evol 2020;85:104438. 32615317 10.1016/j.meegid.2020.104438 PMC7832673

[133] Ryan KA , Bewley KR , Fotheringham SA , et al. Dose-dependent response to infection with SARS-CoV-2 in the ferret model and evidence of protective immunity. Nat Commun 2021;12:81. 33398055 10.1038/s41467-020-20439-y PMC7782478

[134] Kim Y-I , Kim S-G , Kim S-M , et al. Infection and rapid transmission of SARS-CoV-2 in ferrets. Cell Host Microbe 2020;27:704–709.e2. 32259477 10.1016/j.chom.2020.03.023 PMC7144857

[135] Freuling CM , Breithaupt A , Müller T , et al. Susceptibility of raccoon dogs for experimental SARS-CoV-2 infection. Emerg Infect Dis 2020;26:2982–2985. 33089771 10.3201/eid2612.203733 PMC7706974

[136] Munster VJ , Feldmann F , Williamson BN , et al. Respiratory disease in rhesus macaques inoculated with SARS-CoV-2. Nature 2020;585:268–272. 32396922 10.1038/s41586-020-2324-7 PMC7486227

[137] Rockx B , Kuiken T , Herfst S , et al. Comparative pathogenesis of COVID-19, MERS, and SARS in a nonhuman primate model. Science 2020;368:1012–1015. 32303590 10.1126/science.abb7314 PMC7164679

[138] Mykytyn AZ , Lamers MM , Okba NMA , et al. Susceptibility of rabbits to SARS-CoV-2. Emerg Microbes Infect 2021;10:1–7. 33356979 10.1080/22221751.2020.1868951 PMC7832544

[139] Schlottau K , Rissmann M , Graaf A , et al. SARS-CoV-2 in fruit bats, ferrets, pigs, and chickens: an experimental transmission study. Lancet Microbe 2020;1:e218–e225. 32838346 10.1016/S2666-5247(20)30089-6 PMC7340389

[140] Imai M , Iwatsuki-Horimoto K , Hatta M , et al. Syrian hamsters as a small animal model for SARS-CoV-2 infection and countermeasure development. Proc Nat Acad Sci 2020;117:16587–16595. 32571934 10.1073/pnas.2009799117 PMC7368255

[141] Palmer MV , Martins M , Falkenberg S , et al. Susceptibility of white-tailed deer (Odocoileus virginianus) to SARS-CoV-2. *J Virol* 2021. doi: 10.1128/JVI.00083-21. PMC8139686 33692203

[142] Chandler JC , Bevins SN , Ellis JW , et al. SARS-CoV-2 exposure in wild white-tailed deer (*Odocoileus virginianus*). *bioRxiv* 2021. doi: 10.1101/2021.07.29.454326. PMC8617405 34732584

[143] Gryseels S , De Bruyn L , Gyselings R , Calvignac-Spencer S , Leendertz FH , Leirs H. Risk of human-to-wildlife transmission of SARS-CoV-2. *Mamm Rev* 2020. doi: 10.1111/mam.12225. PMC7675675 33230363

[144] Griffin JB , Haddix M , Danza P , et al. SARS-CoV-2 Infections and hospitalizations among persons aged ≥16 years, by vaccination status—Los Angeles County, California, May 1–July 25, 2021. Morb Mortal Wkly Rep 2021;70:1170–1176. 10.15585/mmwr.mm7034e5 PMC8389389 34437525

[145] Del Rio C , Malani PN , Omer SB. Confronting the delta variant of SARS-CoV-2, summer 2021. *JAMA* 2021. doi: 10.1001/jama.2021.14811. 34406361

[146] Lazarevic I , Pravica V , Miljanovic D , Cupic M. Immune evasion of SARS-CoV-2 emerging variants: what have we learnt so far? *Viruses* 2021;13. doi: 10.3390/v13071192. PMC8310325 34206453

[147] Kemp SA , Collier DA , Datir RP , et al. SARS-CoV-2 evolution during treatment of chronic infection. Nature 2021;592:277–282. 33545711 10.1038/s41586-021-03291-y PMC7610568

[148] Srivastava S , Banu S , Singh P , Sowpati DT , Mishra RK. SARS-CoV-2 genomics: an Indian perspective on sequencing viral variants. *J Biosci* 2021;46. doi: 10.1007/s12038-021-00145-7 PMC7895735 33737495

[149] Furuse Y. Genomic sequencing effort for SARS-CoV-2 by country during the pandemic. Int J Infect Dis 2021;103:305–307. 33333251 10.1016/j.ijid.2020.12.034 PMC7832795

[150] Crawford DC , Williams SM. Global variation in sequencing impedes SARS-CoV-2 surveillance. PLoS Genet 2021;17:e1009620. 34264957 10.1371/journal.pgen.1009620 PMC8282079

[151] Adepoju P. Challenges of SARS-CoV-2 genomic surveillance in Africa. Lancet Microbe 2021;2:e139. 33817677 10.1016/S2666-5247(21)00065-3 PMC8009639

[152] Blomberg N , Lauer KB. Connecting data, tools and people across Europe: ELIXIR’s response to the COVID-19 pandemic. Eur J Hum Genet 2020;28:719–723. 32415272 10.1038/s41431-020-0637-5 PMC7225634

[153] Conesa A , Beck S. Making multiomics data accessible to researchers. Sci Data 2019;6:251. 31672978 10.1038/s41597-019-0258-4 PMC6823467

[154] Hodcroft EB , De Maio N , Lanfear R , et al. Want to track pandemic variants faster? Fix the bioinformatics bottleneck. Nature 2021;591:30–33. 33649511 10.1038/d41586-021-00525-x

[155] Turakhia Y , Thornlow B , Hinrichs AS , et al. Ultrafast Sample placement on Existing tRees (UShER) enables real-time phylogenetics for the SARS-CoV-2 pandemic. Nat Genet 2021;53:809–816. 33972780 10.1038/s41588-021-00862-7 PMC9248294

[156] Wenzel J. Origins of SARS-CoV-1 and SARS-CoV-2 are often poorly explored in leading publications. Cladistics 2020;36:374–379. 34618963 10.1111/cla.12425

[157] Machado DJ , Schneider AB , Guirales S , Janies DA. FLAVi: an enhanced annotator for viral genomes of Flaviviridae. *Viruses* 2020;12. doi: 10.3390/v12080892 10.3390/v12080892 PMC7472247 32824044

[158] Wheeler WC. Sequence alignment, parameter sensitivity, and the phylogenetic analysis of molecular data. Syst Biol 1995;44:321–331.

[159] Grant T. The perils of “point-and-click” systematics. Cladistics 2003;19:276–285.

[160] Hovmöller R , Alexandrov B , Hardman J , Janies D. Tracking the geographical spread of avian influenza (H5N1) with multiple phylogenetic trees. Cladistics 2010;26:1–13. 34875747 10.1111/j.1096-0031.2009.00297.x

[161] de Bernardi Schneider A , Ford CT , et al. StrainHub: a phylogenetic tool to construct pathogen transmission networks. Bioinformatics 2020;36:945–947. 31418766 10.1093/bioinformatics/btz646 PMC8215912

[162] Syrowatka A , Kuznetsova M , Alsubai A , et al. Leveraging artificial intelligence for pandemic preparedness and response: a scoping review to identify key use cases. NPJ Digit Med 2021;4:96. 34112939 10.1038/s41746-021-00459-8 PMC8192906

[163] Chen S , Owolabi Y , Li A , et al. Patch dynamics modeling framework from pathogens’ perspective: Unified and standardized approach for complicated epidemic systems. PLoS One 2020;15:e0238186. 33057348 10.1371/journal.pone.0238186 PMC7561140

[164] COVID-19. Johns Hopkins Coronavirus Resource Center website. https://coronavirus.jhu.edu/map.html. Accessed October 12, 2021.

[165] Henderson DA. The eradication of smallpox—an overview of the past, present, and future. Vaccine 2011;29 suppl 4:D7–9. 10.1016/j.vaccine.2011.06.080 22188929

[166] Arunkumar G , Chandni R , Mourya DT , et al. Outbreak investigation of Nipah virus disease in Kerala, India, 2018. J Infect Dis 2019;219:1867–1878. 30364984 10.1093/infdis/jiy612

[167] Soh SM , Kim Y , Kim C , Jang US , Lee H-R. The rapid adaptation of SARS-CoV-2-rise of the variants: transmission and resistance. J Microbiol 2021;59:807–818. 34449057 10.1007/s12275-021-1348-5 PMC8390340

[168] Schwarze K , Buchanan J , Fermont JM , et al. The complete costs of genome sequencing: a microcosting study in cancer and rare diseases from a single center in the United Kingdom. Genet Med 2020;22:85–94. 31358947 10.1038/s41436-019-0618-7 PMC6944636

